# Whole-genome sequence and methylome profiling of the almond [*Prunus dulcis* (Mill.) D.A. Webb] cultivar ‘Nonpareil’

**DOI:** 10.1093/g3journal/jkac065

**Published:** 2022-03-23

**Authors:** Katherine M D’Amico-Willman, Wilberforce Z Ouma, Tea Meulia, Gina M Sideli, Thomas M Gradziel, Jonathan Fresnedo-Ramírez

**Affiliations:** 1 Center for Applied Plant Sciences, The Ohio State University, Wooster, OH 44691, USA; 2 Ohio Supercomputer Center, Columbus, OH 43215, USA; 3 Molecular and Cellular Imaging Center, The Ohio State University, Wooster, OH 44691, USA; 4 Department of Plant Sciences, University of California, Davis, Davis, CA 95616, USA; 5 Department of Horticulture and Crop Science, The Ohio State University, Wooster, OH 44691, USA

**Keywords:** almond, Nonpareil, enzymatic methylation sequencing, cytosine methylation, multiplatform genome assembly

## Abstract

Almond [*Prunus dulcis* (Mill.) D.A. Webb] is an economically important, specialty nut crop grown almost exclusively in the United States. Breeding and improvement efforts worldwide have led to the development of key, productive cultivars, including ‘Nonpareil,’ which is the most widely grown almond cultivar. Thus far, genomic resources for this species have been limited, and a whole-genome assembly for ‘Nonpareil’ is not currently available despite its economic importance and use in almond breeding worldwide. We generated a 571X coverage genome sequence using Illumina, PacBio, and optical mapping technologies. Gene prediction revealed 49,321 putative genes using MinION Oxford nanopore and Illumina RNA sequencing, and genome annotation found that 68% of predicted models are associated with at least one biological function. Furthermore, epigenetic signatures of almond, namely DNA cytosine methylation, have been implicated in a variety of phenotypes including self-compatibility, bud dormancy, and development of noninfectious bud failure. In addition to the genome sequence and annotation, this report also provides the complete methylome of several almond tissues, including leaf, flower, endocarp, mesocarp, exocarp, and seed coat. Comparisons between methylation profiles in these tissues revealed differences in genome-wide weighted % methylation and chromosome-level methylation enrichment.

## Introduction

In the past decade, significant advances have been made in the ability to produce whole-genome sequences from *Prunus* species. Challenges due to the inherent characteristics of outcrossing heterozygous plants have prevented the *Prunus* community from producing high-quality assemblies using second-generation high-throughput sequencing (Illumina) and pyrosequencing (Roche 454) technologies. As a result, the first *Prunus* genome was produced using a doubled-haploid of the peach rootstock genotype ‘Lovell’ (The International Peach Genome Initiative *et al.* 2013). Although this ‘Lovell’ genome was further refined using short-read sequencing data to ensure continuity and completeness (Verde *et al.* 2017), the capabilities for developing genome assemblies for heterozygous *Prunus* genotypes were very limited. These limitations excluded other commercially relevant cultivars from being sequenced at that time.

The recent advent, improvement, and successful implementation of third-generation high-throughput sequencing technologies such as single-molecule real-time sequencing (Pacific Biosciences) and Nanopore Sequencing (Oxford) have been key to sequencing heterozygous *Prunus* genomes. At the time of preparing this manuscript, 20 genomes assemblies for 13 *Prunus* species (https://www.rosaceae.org/species/prunus/all, last accessed 2.2.2022) are reported in the Genome Rosaceae Database (GDR, www.rosaceae.org, last accessed 2.2.2022; Jung *et al.* 2019). Among these new resources are 2 *Prunus* *dulcis* genomes: one from the cultivar ‘Lauranne’ (Sánchez-Pérez *et al.* 2019), which was used to investigate kernel bitterness, and the other from the cultivar ‘Texas’ (a.k.a. ‘Mission’; Alioto *et al.* 2020), which was used to study the nature of transposable elements and their contribution to structural divergence from peach. Additional work has been done in *Prunus* to examine the impact of DNA methylation on phenotypes such as dormancy and fruit quality traits (Rothkegel *et al.* 2017; Prudencio *et al.* 2018; García-Gómez *et al.* 2020).

Since 2018, our group has worked to produce a genome assembly for ‘Nonpareil,’ the most widely grown almond genotype. ‘Nonpareil’ was first described in 1879 and currently represents 42% of US almond production along with significant use in other almond-producing countries such as Australia (Almond Board of California 2020). ‘Nonpareil’ is considered the reference cultivar in terms of field performance and kernel quality for a large portion of the industry and is therefore used as a recurrent parent in almond breeding programs (see Supplementary File 1 for a brief pomological description of Nonpareil and the additional almond cultivars with sequenced genome). However, ‘Nonpareil’ is susceptible to the aging-related disorder noninfectious bud failure (BF), which can negatively impact kernel yield and has been associated with genome-wide DNA methylation (Fresnedo-Ramírez *et al.* 2017). Our group developed a genome assembly as well as methylomes for a variety of almond tissues using this relevant cultivar to address BF by assessing the risk of onset in almond breeding material. Developing and exploring *Prunus* genomes such as ‘Nonpareil’ will shed light on the genome polymorphism and dynamism involved in the exhibition of agriculturally relevant traits and syndromes.

Therefore, the primary objectives of our work are:

1. To provide a robust, complete genome sequence of the most important almond cultivar, ‘Nonpareil.’
2. To provide methylomes of ‘Nonpareil’ tissues: including leaf, flower, endocarp, mesocarp, exocarp, and seed coat which are of interest to our group in research focused on DNA methylation and its impact on aging-related disorders.
3. To provide the first ‘Nonpareil’ genome suite, which includes complete assembly of the nuclear, plastidial, and mitochondrial genomes.

In summary, the goal of this genome report is to present additional, high-quality genomic tools to the scientific community to enable research in almond and related species.

## Materials and methods

### Plant material

Tissue samples for the genome sequencing and methylome profiling were collected in 2018, 2019, and 2020 from a single ‘Nonpareil’ clone: GOH B32 T37-40 maintained at Foundation Plant Services—University of California, Davis (Davis, CA, USA). This ‘Nonpareil’ clone is recognized as the foundational source for any ‘Nonpareil’ almond deployed in commercial orchards worldwide. Leaf samples were collected in 2018 for whole-genome and RNA sequencing from another ‘Nonpareil’ clone located at the Wolfskill Experimental Orchards—University of California, Davis (Winters, CA, USA), which is grafted onto ‘Nemaguard’ peach rootstock and exhibits noninfectious BF.

Initially, leaf samples were collected for whole-genome and RNA sequencing in 2018. In 2019, additional leaf samples were collected for optical mapping, while phloem tissue and fruits were collected for DNA methylation profiling and RNA sequencing. In 2020, flower tissues were collected for RNA sequencing and DNA methylation profiling prior to anthesis. Leaf tissues were collected on ice in the field and stored at −20°C until sample processing, while flower tissue was collected and immediately put on dry ice and stored at −80°C until processing. Leaf samples collected in 2018 and 2019 were processed at UC Davis, and additional samples were shipped overnight on dry ice in 2018 to the Ohio Agricultural Research and Development Center (OARDC—Wooster, Ohio) and stored at −40°C until processing for RNA sequencing. Fruits and phloem tissues collected in 2019 were shipped overnight on ice to the OARDC and stored at −20°C until sample processing. Flower tissues were processed at UC Davis for RNA sequencing, and additional samples were shipped overnight on ice to the OARDC for DNA methylation profiling.

### DNA isolation

For long-read and short-read sequencing, leaf samples were sent to Dovetail Genomics, where DNA was isolated using a standard CTAB protocol. For optical mapping, ultra-high molecular weight (uHMW) DNA was isolated from leaves using the Plant DNA Isolation Kit (Bionano Genomics, San Diego, CA, USA) according to the manufacturer’s instructions. The uHMW DNA molecules were labeled with the DLE-1 enzyme (Bionano Genomics, San Diego, CA, USA) and stained using the Bionano Prep Direct Label and Stain (DLS) Kit (Bionano Genomics, San Diego, CA, USA) according to the manufacturer’s instructions.

For methylation profiling, DNA was isolated from young leaves, fruits, and flowers. To extract DNA from fruit, the fruits were first dissected using a scalpel while frozen to isolate exocarp, mesocarp, endocarp, and seed coat tissues. All tissues were then ground in liquid nitrogen using mortar and pestle, and 150 mg of ground material was used as input into the SILEX DNA isolation protocol outlined in Vilanova *et al.* (2020) with some modifications. To isolate DNA from young leaves, leaf tissue was ground to a fine powder in liquid nitrogen using a mortar and pestle, and 150 mg of ground material was used as input into the SILEX DNA isolation protocol as above. Finally, DNA was isolated from whole flowers using the same method of grinding in liquid nitrogen and isolating following the SILEX protocol. All isolated DNA was assessed for concentration and quality by fluorometry and electrophoresis using a Qubit 4 and Qubit 1X dsDNA HS Assay Kit (ThermoFisher Scientific) and a TapeStation (Agilent, Santa Clara, CA, USA).

### RNA isolation, library preparation, and sequencing

To isolate RNA from leaf, fruit, and phloem tissues samples, tissue was ground in liquid nitrogen using a mortar and pestle, and 50 mg of tissue was used as input into the RNA isolation protocol outlined in Gambino *et al.* (2008). To isolate RNA from flowers, the tissues were ground in liquid nitrogen, and the ground material was used as input in ThermoFisher Scientific PureLink Plant RNA Reagent. Following extraction, RNA was DNase treated using the DNA-*free* DNA Removal Kit (ThermoFisher Scientific) according to the manufacturer’s instructions. RNA concentration and quality were assessed by fluorometry and electrophoresis using a Qubit 4 and Qubit RNA HS Assay Kit (ThermoFisher Scientific) and a TapeStation (Agilent).

A sequencing library for the flower RNA sample was prepared using the NEBNext Ultra II Directional RNA Library Prep Kit for Illumina (New England BioLabs, Inc., Ipswich, MA, USA) and barcoded using index primers from the NEBNext Multiplex Oligos for Illumina (New England BioLabs) following the manufacturer’s instructions. The library was equimolarly pooled and split for sequencing on 2 lanes of the Illumina HiSeq 4000 in paired-end 2 × 150-bp mode.

The RNA-seq libraries for short-read sequencing for the fruit, leaf, and phloem tissues were also prepared using the NEBNext Ultra II Directional RNA Library Prep Kit for Illumina (New England BioLabs) and barcoded using index primers from the NEBNext Multiplex Oligos for Illumina (New England BioLabs). These libraries were processed using the MiSeq Reagent Kit v2 according to the manufacturer's protocols and were sequenced in an Illumina MiSeq device in paired-end 2 × 150-bp mode.

### Sequencing of RNA-seq libraries using Oxford Nanopore Technology

Total RNA from leaves was first depleted using the Illumina Ribo-Zero rRNA Removal Kit (Plant). The RNA was then purified and concentrated using the RNA Clean Concentrator-5 kit (Zymo Research, Irvine, CA, USA). cDNA libraries were prepared using a mix of 50 ng RNA and 0.5 ng Spike-in RNA Variant Control Mix E2 (Lexogen, NH, USA) according to the Oxford Nanopore Technologies (Oxford Nanopore Technologies Ltd, Oxford, UK) protocol “DNA-PCR Sequencing” with 14 cycles of PCR (8-min elongation time). Oxford Nanopore Technology adapters were ligated to 650 ng of cDNA. These libraries were sequenced using a MinION Mk1b with an R9.4.1 flowcell. The data were preprocessed using MinKNOW 3.1.18, and the base calling was done using Guppy 2.0.5.

### Library construction, sequencing, and assembly of PacBio data

Long-read sequencing was performed with a PacBio Sequel II System (Pacific Biosciences, Menlo Park, CA, USA) using Single Molecule, Real-Time (SMRT) technology. Genomic DNA libraries were prepared with 5 µg of input DNA according to the “Guidelines for Preparing 20 kb SMRTbell™ Templates” (available at https://www.pacb.com/wp-content/uploads/2015/09/User-Bulletin-Guidelines-for-Preparing-20-kb-SMRTbell-Templates.pdf, last accessed 2.2.2022). Sequencing was performed on one PacBio Sequel II 8M SMRT cell by Dovetail Genomics (Santa Cruz, CA, USA). The data yielded from the long-read sequencing was processed for de novo assembly using the FALCON v. 0.3.0 (Chin *et al.* 2016) pipeline customized by Dovetail Genomics for the assembly of a heterozygous genome. The assembly was polished using Arrow v. 2.3.3 (available at https://github.com/PacificBiosciences/pbbioconda, last accessed 2.2.2022).

### Optical map construction

A consensus optical map was assembled de novo with the assembler tool in the Bionano Solve v. 3.4 package (available at https://bionanogenomics.com/support/software-downloads/, last accessed 2.2.2022) using significance cutoffs of *P* < 1 × 10^−8^ to generate draft consensus contigs, *P* < 1 × 10^−9^ for draft consensus contig extension, and *P* < 1 × 10^−15^ for the final merging of the draft consensus contigs; a recipe of “haplotype,” “noES,” and “noCut” was chosen for the assembly. The initial optical map was then checked for potential chimeric contigs and further refined.

### Scaffolding

The sequence assembly was validated by comparing it with the optical map. The contigs of the PacBio assembly were digested in silico with DLE-1 restriction sites using Knickers (Bionano Genomics, San Diego, CA, USA). The alignment was performed using the RefAligner tool in the Bionano Solve v 3.4 package (Bionano Genomics, San Diego, CA, USA) with an initial alignment cutoff of *P* < 1 × 10^−10^. The PacBio contigs that disagreed with the optical map were disjoined accordingly, and the conflict-free contigs were linked with the guide of the optical map. The gaps were then filled with the respective number of N’s using the estimated length between the flanking restriction sites.

### Pseudomolecule construction

Genotyping-by-sequencing (GBS) data generated for a mapping population of 89 individuals from a cross between the cultivars ‘Nonpareil’ and ‘Lauranne’ (Goonetilleke *et al.* 2018) were used to produce a linkage map using the software package RABBIT v. 3.2. (Zheng *et al.* 2019; https://github.com/chaozhi/RABBIT, last accessed 2.2.2022). The GBS data were retrieved from the European Nucleotide Archive (accession number PRJEB23106) and was processed using the TASSEL v. 5.2.7.1 pipeline (Glaubitz *et al.* 2014) to call for single nucleotide polymorphisms (SNPs) with the first iteration of the ‘Nonpareil’ assembly generated from optical mapping. These SNPs were filtered using a minor allele frequency of 0.1 and no missingness. These parameters were chosen to keep at least 3 segregating SNPs per scaffold to link to a linkage group. The resulting SNPs were then coded and processed with RABBIT in a linkage map representing the 8 main linkage groups. This linkage map, along with the assembly and gene annotations of *P.* *dulcis* cv. ‘Texas’ v. 2.0 (Alioto *et al.* 2020), was used to validate the general structure, order, and orientation of the produced pseudomolecules. The ‘Lauranne’ cultivar assembly was not used because this assembly is fragmented and incomplete (see *Results and Discussion*). The linkage map alone was then used to determine the order and orientations of the scaffolds on each chromosome. The scaffolds were linked with 100 N’s accordingly and anchored onto the 8 chromosomes.

Finally, for the organelles, short-read genomic data and the GetOrganelle v. 1.7.5.3 pipeline (Jin *et al.* 2020) were used along with the almond plastid (NCBI Reference Sequence: NC_034696.1) and cherry mitochondrion (GenBank: MK816392.2) to guide the draft assemblies. The final assemblies were integrated into the final fasta file, including the 8 pseudomolecules of the nuclear genome, the 2 organelle scaffolds, and the unanchored contigs. After submission to GenBank, putative mitochondrial contaminants in the nuclear genome were eliminated prior to the final deposit of the assembly.

### Gene prediction and annotation

Prior to gene prediction, repetitive elements were identified, and the genome assembly was masked using the Extensive de novo TE Annotator (EDTA) v. 2.0.0 pipeline (Ou *et al.* 2019) and the PGSB Repeat Element Database for Eudicots v. 9.3 (Nussbaumer *et al.* 2013). Transcriptome assembly was performed in hybrid mode (Antipov *et al.* 2016) with short- and long-read RNA-seq data using SPAdes v. 3.1.5.3 (Prjibelski *et al.* 2020). Gene prediction was performed using the BREAKER2 v. 2.1.6 (Brůna *et al.* 2021) pipeline with the –etpmode option (Brůna *et al.* 2020) along with the masked genome produced by EDTA. Also used as input for gene prediction was a BAM file produced when merging the short-read alignment from STAR v. 2.7.9a (Dobin *et al.* 2013) and the long-read alignment from Minimap2 v. 2.23 (Li 2018): both produced using the masked genome and reference proteome for *P.* *dulcis* hosted in UniProt (accession UP000327085) and curated in Alioto *et* * al.* (2020). The gene prediction from BREAKER2 was subsequently processed through the PASA pipeline v. 2.5.1 (Haas *et al.* 2003) using the hybrid transcriptome produced in SPAdes to analyze spliced alignments and produce gene structures. The GFF3 files produced from BREAKER2 and PASA were subsequently analyzed through EVidenceModeler v. 1.1.1 (Haas *et al.* 2008) to combine ab initio gene predictions from BREAKER2 and protein transcript alignments from PASA to produce a final gene prediction and annotation file.

For functional annotation, predicted gene models were submitted to the InterProScan v. 5.54 pipeline utilizing database searches of protein sequences in Uniprot (homology search; The UniProt Consortium 2019), KEGG database (orthology search; Kanehisa *et al.* 2008), and Pfam (The UniProt Consortium 2019). The resulting descriptions of putative gene functions, Pfam domain identifiers, and gene ontology terms were included in the genome annotation feature file.

Annotation of the plastid sequences was performed using the beta version of the web service Chloë (https://chloe.plantenergy.edu.au, last accessed 2.2.2022; Zhong and University of Western Australia 2020), and annotation of the mitochondrion sequence was performed using GeSeq (Tillich *et al.* 2017) hosted in MPI-MP CHLOROBOX (https://chlorobox.mpimp-golm.mpg.de/geseq.html, last accessed 2.2.2022).

### Genome assembly assessment

Analysis of metrics based on evolutionarily informed expectations of gene content of near-universal single-copy orthologs (BUSCO, Manni *et al.* 2021) for final genome assembly, transcriptome, and proteome was performed using the BUSCO v. 5.2.2 pipeline and the BUSCO dataset for Eudicots v. 10 (eudiccots_odb10). Datasets from the almond genome sequences for ‘Lauranne’ (Sánchez-Pérez *et al.* 2019), ‘Texas’ (Alioto *et al.* 2020), and the peach reference genome produced from the genotype ‘Lovell’ (Verde *et al.* 2017) were used as references for this study.

### Enzymatic methyl-seq library preparation and sequencing

Whole-genome enzymatic methyl-seq libraries were prepared using the NEBNext Enzymatic Methyl-seq kit (New England BioLabs, Inc.) following the protocol for standard insert libraries (370–420 base pairs). Each sample was prepared using 100 ng input DNA in 48 µL TE buffer (1 mM Tris-HCl; 0.1 mM EDTA; pH 8.0) with 1 µL spikes of both the CpG unmethylated Lambda and CpG methylated pUC19 control DNA provided in the kit. The samples were sonicated using a Covaris S220 focused-ultrasonicator in microTUBE AFA Fiber Pre-Slit Snap-Cap 6 × 16 mm tubes (Covaris, Woburn, MA, USA) with the following program parameters: peak incident power (W) = 140; duty factor = 10%; cycles per burst = 200; treatment time (s) = 80.

Following library preparation, library concentration and quality were assessed by fluorometry using a Qubit 4 and Qubit 1X dsDNA HS Assay Kit (ThermoFisher Scientific) and by electrophoresis using a TapeStation (Agilent). Library concentration was further quantified by qPCR using the NEBNext Library Quant Kit for Illumina (New England BioLabs, Inc.). Libraries were sequenced on one lane of the Illumina HiSeq4000 platform to generate 150-bp paired-end reads.

### Processing and alignment of enzymatic methyl-seq libraries

Methyl-Seq read quality was initially assessed using FastQC v. 0.11.7 (Andrews 2010), and reads were trimmed using TrimGalore v. 0.6.6 and Cutadapt v. 2.10 with default parameters (Krueger 2016). Forward read fastq and reverse read fastq files from the 2 HiSeq4000 lanes were combined for each library to produce single fastq files for both read one and read two. Reads were aligned to the ‘Nonpareil’ v. 2.0 almond reference genome, deduplicated, and methylation calls were generated using Bismark v. 0.22.3 (Krueger and Andrews 2011) with default parameters in paired-end mode. Reads were also aligned to both the Lambda and pUC19 nucleotide sequence fasta files provided by NEB (https://www.neb.com/tools-and-resources/interactive-tools/dna-sequences-and-maps-tool) to test conversion efficiency. All analyses were performed using the Ohio Supercomputer Center computing resources (Ohio Supercomputer Center, 1987).

### Analysis of DNA methylation profiles in almond tissues

Weighted genome-wide % methylation values were calculated for each tissue type by taking the total number of methylated reads at each cytosine and dividing this by the total number of reads (methylated + unmethylated) at each cytosine. Weighted values were calculated for each methylation context. Methylation call files were then subset by chromosome (chr1—chr8), and weighted % methylation values were calculated for each tissue type by chromosome using the same formula as above for each methylation context. Circos plots were generated to visualize weighted % methylation across the 8 chromosomes in the ‘Nonpareil’ genome, with one track depicting the weighted % methylation for each of 5 tissue types (leaf, exocarp, mesocarp, endocarp, and seedcoat) across the genome. The circos plots were created with the R package circlize v. 0.41.2 (Gu *et al.* 2014), along with text files containing aggregated % methylation values generated using bedtools *makewindows* with a bin size of 100,000 base pairs (Quinlan and Hall 2010). The command *circos.genomicTrack()* was used to create each track depicting weighted % methylation for each tissue type across the 8 chromosomes of the almond genome (Gu *et al.* 2014).

## Results and discussion

### Nonpareil genome assembly

The first version of the almond ‘Nonpareil’ genome assembly (available at: https://www.rosaceae.org/rosaceae_downloads/Prunus_dulcis/Nonpareil_v1.tar.gz, last accessed 2.2.2022) was the result of a combination of Illumina technology (HiSeq X) and Hi-C data in the form of CHiCAGO and Dovetail HiRise implementations. With a coverage of 3739.9X [assuming a genome size of 240 Mb estimated by flow cytometry in 2018 prior to Sánchez-Pérez *et al.* (2019) and Alioto *et al.* (2020) studies], the resulting assembly of 164.55 Mb represented the gene space of ‘Nonpareil’ assembled in 2,081 scaffolds with N50 = 15.28 kb. Using Hi-C, it was possible to infer the 8 major pseudomolecules expected in the haploid assembly representing the genome space of ‘Nonpareil.’

Here, we present the results of a second iteration of the assembly and genome annotation of ‘Nonpareil.’ The purpose was to improve the representation, completeness, and orientation of the genome using long-read sequencing coupled with optical mapping technology. Approximately 793 million reads were produced from PacBio CLR libraries sequenced on the PacBio Sequel II, yielding ∼147 Gb of data with a mean read length of 23.3 kb. These reads represent a coverage of ∼571X (assuming a genome size of 257.2 Mb as our current estimate). A total of 784.27 million corrected reads were used as input in the PacBio FALCON whole-genome assembly pipeline. The N50 length of the error corrected reads was 29.33 kb. The resulting assembly had a contig N50 of 1.34 Mb and N90 of 384.64 kb. The final number of polished contigs was 593 encompassing 456.66 Mb with a 97.1% BUSCO completeness score and an NG50 of 2.83 Mb.

This assembly was used as input for optical mapping for which ∼52 Gb of large single molecules (>150 Kb), labeled by DLE-1 enzyme (Bionano Genomics), were collected and de novo assembled into an optical map. This map consists of 227 contigs with a total length of 533.72 Mb and an N50 of 3.19 Mb. We aligned the 456.66 Mb PacBio contigs (Contig_v1.0; Table 1) of almond to the optical map and identified 244 conflicts included in 194 contigs (totaled 288,407,142 bp). These chimeric contigs were resolved, and the redundancies were removed. The conflict-free dataset (Contig_v1.1) consists of 740 contigs with a slightly decreased N50 size of 1,308,716 bp (Table 1). The sequences of Contig_v1.1 were used to generate scaffolds with the optical map as a guide resulting in scaffolds with a total length of 458,275,742 bp and N50 size of 3,168,198 bp (Scaffold_all; Table 1). The longest scaffold size was 27,216,209 bp.

**Table 1.** Summary of scaffolding using the optical map.

	Contig_v1.0	Contig_v1.1	Scaffold_all	Scaffold_hap
No. of sequences	593	740	499	50
Max length	6,727,137	6,081,999	27,216,209	27,216,209
Total size	456,658,006	455,350,334	458,275,742	254,451,385
Sequence N50	1,838,609	1,308,716	3,168,198	9,199,899
*N*%	0	0	0.37%	0.40%

The optical map was self-aligned to obtain 2 datasets, each representing a haplotype. As a result, 2 datasets with total sizes of ∼275 and 258 Mb, respectively, were generated. The Scaffold_all was divided into 2 datasets accordingly by aligning sequences with the 2 sets of optical maps. The primary scaffold set (Scaffold_hap; Table 1), which has a total size of 254,451,385 bp and an N50 of 9,199,899, was used to construct pseudomolecules since ∼275 Mb was highly redundant and chimeric.

To orient the pseudomolecules, 9,593 SNPs were selected for linkage map construction, and 7,051 were placed in the resulting linkage map. This number of SNPs placed in the linkage exceeded the expectation of having more than 3 markers per scaffold; thus, we anchored all 50 scaffolds of Scaffold_hap onto the 8 expected chromosomes, though the 95 additional contigs not anchored through the optical mapping were also not anchored through the linkage map. Validation of the structure and order of the ‘Nonpareil’ assembly with annotated sequences (genes) in the ‘Texas’ genome was assessed visually.

This report provides the first draft plastid and mitochondrion assemblies for almond, contributing to this limited genomic resource in plants. These prokaryotic genomes are part of the genome suite of all plants but are usually neglected in plant genome sequencing, assembly, and annotation projects. Although the NCBI entry for the almond reference genome (‘Texas’) shows the plastid, this plastid sequence was actually produced by another group and was not part of the Alioto *et al.* (2020) study.

Here, we provide draft sequences and diagrams for both organellar genomes (Supplementary Fig. 1 for plastid and Supplementary Fig. 2 for mitochondrion). These draft sequences were generated using the short-read data only since many software packages do not provide support for the assembly of organellar genomes using long-read sequencing technology. Though short-read sequencing may be sufficient for generating representative plastid genomes, this is not the case for mitochondria, which have proven challenging to generate and characterize assemblies for (Kozik *et al.* 2019; Jin *et al.* 2020). The objective of providing these 2 draft sequences is to enable research on these organelles, which up to now has been limited. A simple application of this resource might be pedigree reconstruction since (as far as we know) mitochondria and plastids are maternally inherited. Thus, polymorphisms identified in these organelles (more often in mitochondria than in plastid typically) may aid in the accurate determination of parentages in breeding or natural populations, as has been done using the self-incompatibility alleles (Pérez De Los Cobos *et al*. 2021).

**Fig. 1.**
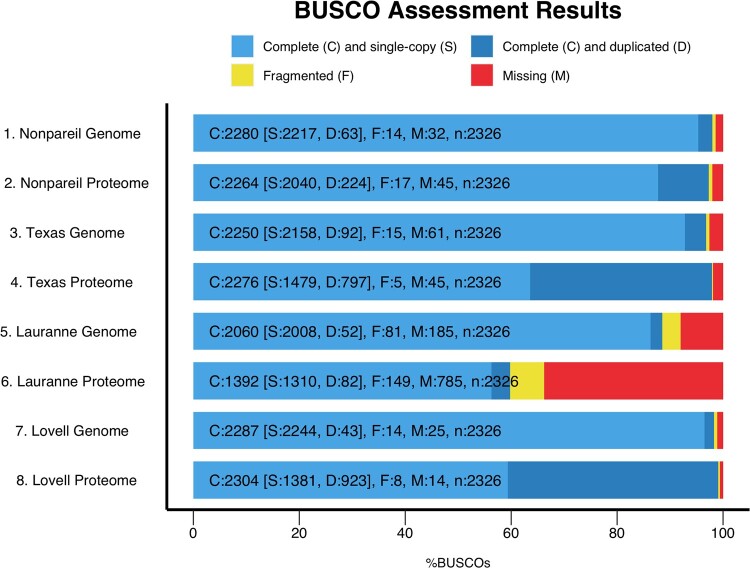
BUSCO metrics graphical representation for genome assembly and proteome, respectively, for almond cultivars ‘Nonpareil,’ ‘Texas,’ and ‘Lauranne,’ in reference to the metrics from the peach reference genome sequence version 2.0.1a for the cultivar ‘Lovell.’

The general quantitative overview of the whole-genome suite of ‘Nonpareil’ is summarized in Table 2. The BUSCO scores for the final assembly (see Fig. 1 for graphical representation and comparison) were 98% for complete BUSCOs, 0.6% fragmented, and 1.4% missing. The N50 was 1,748,356, L50: 49, and GC%: 37.99. In general, the BUSCO scores are comparable to those from ‘Texas’ (N50 = 115,182, L50: 511), which is considered the reference, and far superior to those from ‘Lauranne’ (N50 = 82,269, L50: 791), which in general is an incomplete and fragmented genome. It is worth mentioning that the goal of the ‘Lauranne’ genome sequence, however, was not to study the genome but to develop a tool that allowed a deeper investigation of the bHLH transcription factor influencing kernel sweetness. In terms of GC%, the values are similar (‘Texas’ = 37.85, ‘Lauranne’= 37.9), which is expected since the assemblies come from the same species. It is notable that the quality of these genomes is very high despite being developed from heterozygous individuals (as is common in almond germplasm). When compared with the peach reference genome (‘Lovell,’ Fig. 1), the almond genomes (especially ‘Nonpareil’ and ‘Texas’) show comparable quality and completeness considering the peach genome was derived from a doubled-haploid individual.

**Table 2.** Summary of the sequences of the genomes of ‘Nonpareil.’

Chromosome	Length (bp)	Effective length (bp)	*N*%
Pdu1	53,037,566	52,621,683	0.78
Pdu2	33,982,222	33,650,668	0.98
Pdu3	30,537,282	30,345,236	0.63
Pdu4	30,643,767	30,642,693	0.01
Pdu5	21,032,966	21,019,937	0.06
Pdu6	32,793,846	32,733,303	0.18
Pdu7	26,338,084	26,325,646	0.05
Pdu8	26,045,089	26,041,993	0.01
Additional contigs	2,660,941	2,660,941	0.00
*Total nuclear*	*257,071,763*	*256,042,100*	*0.30*
Plastid	142,856	142,854	0.00
Mitochondrion	444,092	444,092	0.00
Grand total	257,658,711	256,629,046	0.30

Interestingly, the ‘Nonpareil’ assembly showed a larger genome size than expected, particularly when compared with ‘Texas’ (227.76 Mb) and ‘Lauranne’ (246.12 Mb) (https://www.ncbi.nlm.nih.gov/genome/browse/#!/eukaryotes/10947/, last accessed 2.2.2022). Variation in genome size within species and even populations has been observed in distinct eukaryotic species (Stelzer *et al.* 2019), and many factors influence this variation, from actual genomic features such as repetitive elements (Wang *et al.* 2021) to artifacts related to the technologies used for genome assembly (Minio *et al.* 2019). Metrics such as BUSCO in this study provide support of the completeness of the assembly, which represents the closest genome model for almond.

In terms of transcriptome completeness (comparing ‘Texas,’ ‘Lovell,’ and ‘Nonpareil,’ as ‘Laureanne’ does not have a reported transcriptome), the ‘Nonpareil’ transcriptome scored 96.5% complete in BUSCO metrics (56.3% of these are duplicated), 1.4% fragmented, and 2.1% missing. In comparison, ‘Texas’ scored 96.8% complete BUSCO, 0.6% fragmented, and 2.6% missing. Scores for both ‘Nonpareil’ and ‘Texas’ are comparable to those for ‘Lovell,’ with 99.1% complete BUSCO, 0.4% fragmented, and 0.5% missing (45). This trend is similar for the proteome (Fig. 1), for which the 3 almond genome assemblies can be compared. These results suggest that both the ‘Texas’ and ‘Nonpareil’ genome projects lost similar transcripts and, therefore, proteins based on the biology and management of species such as almond. These similarities could be due to the tissues sampled, which do not include tissues like root due to complications in obtaining true roots from grafted scions. It is possible that a slight improvement in transcriptome and proteome completeness (and therefore in gene prediction and annotation) can be accomplished if missing tissues (such as root) are sampled and characterized in future efforts as has been done in peach (Verde *et al.* 2017).

The development of the 3 almond genome assemblies included the use of 2 sequencing technologies: short- and long-read sequencing. Assembly refinement required the use of additional technologies, including optical mapping for ‘Nonpareil’ and resources like linkage maps, to enable the production of a high-quality genome despite biological constraints such as heterozygosity.

### Repetitive elements in ‘Nonpareil’

The 3 current almond genome assemblies share a similar proportion of repetitive elements in their genome structure: 34.5% for ‘Lauranne,’ 38.2% for ‘Texas,’ and 33.61% for ‘Nonpareil’ (Table 3), despite distinct pipelines being used to identify and annotate repetitive regions for each cultivar. Repetitive element identification in ‘Lauranne’ (Sánchez-Pérez *et al.* 2019) was performed using RepeatMasker (Smit *et al.* 2013–2015), which is a method based primarily on sequence homology using curated information from a database (knowledge-based approach). For the ‘Texas’ genome, Alioto *et al.* (2020) used TEdenovo and TEannot within the REPET pipeline (Quesneville *et al*. 2005; Flutre *et al*. 2011). These packages emphasize a de novo discovery approach for the identification of repetitive regions and transposable elements within the genome assembly (de novo approach).

**Table 3.** Transposable element composition of the ‘Nonpareil’ genome.

Class	Count	Bp masked	% Masked
LTR—Copia	10,221	7,448,079	2.90
LTR—Gypsy	22,245	20,186,500	7.87
LTR—unknown	24,658	14,319,765	5.58
TIR—CACTA	22,403	10,089,917	3.93
TIR—Mutator	40,465	10,846,793	4.23
TIR—PIF_Harbinger	15,287	5,579,626	2.17
TIR—Tc1 Mariner	1,564	349,110	0.14
TIR—hAT	9,151	3,118,210	1.22
nonLTR—LINE element	593	272,476	0.11
ninLTR—unknown	225	129,418	0.05
nonTIR—helitron	7,240	1,892,602	0.74
Repeat region	37,622	12,025,696	4.69
Total interspersed	191,674	86,258,192	33.61

**Table 4.** Genome-wide weighted percent methylation values for each tissue type in ‘Nonpareil.’

Tissue type	% CG	% CHG	% CHH
Leaf	45.2	14.6	1.3
Flower	56.4	29.4	2.9
Exocarp	52.5	17.7	4.0
Mesocarp	52.9	18.3	4.7
Endocarp	51.7	17.7	4.3
Seed coat	50.5	16.7	4.3

Weighted percent methylation is depicted for each methylation context [CG, CHG, and CHH (H = A, T, or C)].

In our study, repetitive elements were identified with the EDTA pipeline (Ou *et al.* 2019), which uses a hybrid approach, performing automated de novo discovery and annotation of transposable elements and comparing with a collection of curated transposable element sequences. Each method has weaknesses and strengths, some of which are discussed in Ou *et al.* (2019); therefore, a comprehensive comparison of repetitive element composition between the 3 almond assemblies would require a specific experimental design outside the scope of this genome report. However, a summary of trends observed in the reported data is provided below.

The most prevalent type of transposable element identified was the class long terminal repeat (LTR) with 11.1% in ‘Lauranne,’ 21.28% in ‘Texas,’ and 16.35% in ‘Nonpareil.’ Within the LTR class, Copia and Gypsy types were the most common; however, while ‘Lauranne’ and ‘Nonpareil’ contain thousands of copies of these 2 elements (‘Lauranne’:10,421 Copia and 17,665 Gypsy; ‘Nonpareil’: 10,221 Copia and 22,245 Gypsy) (Table 3), ‘Texas’ contains fewer (964 Copia and 325 Gypsy). Trying to compare the estimations other transposable elements from distinct orders/subclasses or types becomes more difficult because several are missed by certain pipelines; thus, for ‘Lauranne,’ terminal inverted repeats (TIRs) are not explicitly counted, but for ‘Texas,’ TIRs represent the second more prevalent transposable element type (13%), like ‘Nonpareil’ (12.69%). As mentioned before, the discrepancy in these numbers may be due to differences in the approaches used to identify, account for, and annotate transposable elements in each study. The similarities observed between ‘Lauranne’ and ‘Nonpareil’ estimations could be because the EDTA pipeline uses RepeatMasker in the final annotation of transposable elements.

Transposable elements are instrumental in genome structure (e.g. genome size) and gene expression and can influence high-order phenomena such as the evolution of almond, as discussed in Alioto *et al.* (2020). There is much more to explore in terms of how transposable elements may influence almond phenotypes through mechanisms such as DNA methylation.

### Nonpareil methylome

Using the genome generated in this report, we analyzed whole-genome DNA methylation profile data for 6 tissue types (leaf, flower, exocarp, mesocarp, endocarp, and seed coat) from ‘Nonpareil.’ To produce the DNA methylation data presented in this paper, we used an enzymatic methyl-seq approach that utilizes enzyme-based conversion of unmethylated cytosines rather than chemical conversion via bisulfite treatment. This method has been shown to have a conversion efficiency equal to or greater than bisulfite conversion (Feng *et al.* 2020) and has been previously used on almond × peach interspecific hybrids to profile DNA methylation (D’Amico-Willman *et al.* 2022). Improved mapping efficiencies using the enzymatic methyl-seq method ranged from 49.7% to 55.7% in this study.

Based on our analysis, we found genome-wide weighted total % methylation values for each methylation context [CG, CHG, CHH (H = C, T, or A); Table 3]. Total % weighted methylation is lowest in the CHH context, as has been reported in other angiosperms (Niederhuth *et al.* 2016). Some variations in genome-wide methylation values were observed across tissue types, with leaf tissue exhibiting the lowest overall methylation levels of the tissues tested. Interestingly, flower tissue showed the highest methylation levels in the CG and CHG context but was observed to have the second-lowest methylation level in the CHH context (Table 4).

**Fig. 2.**
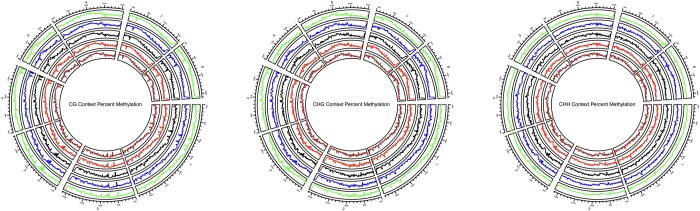
Circos plots depicting genome-wide weighted methylation across each of the 8 almond chromosomes for each methylation context [CG, CHG, and CHH (H = A, T, or C)]. Five almond tissue types are represented in concentric genomic tracks from outer to inner: Leaf, Exocarp, Mesocarp, Endocarp, and Seedcoat.

Genome-wide methylation was also displayed across the 8 chromosomes of the almond genome (Fig. 2) in each of the 3 methylation contexts (CG—Fig. 2a; CHG—Fig. 2b; CHH—Fig. 2c). The circos plots show similar patterns in the distribution of methylation across the almond genome for each tissue type tested (Fig. 2, a–c). Variations in methylation profiles across tissue types in plants have been previously found to be low, particularly in nonembryonic tissues (Schmitz *et al.* 2013; Kawakatsu *et al.* 2016). The generation and availability of methylomes in almond and other related species will be vital to continued breeding and improvement efforts as we continue to realize the importance of DNA methylation in the expression of key traits, as has been shown already in traits such as dormancy (Rothkegel *et al.* 2017; Prudencio *et al.* 2018; García-Gómez *et al.* 2020).

The ‘Nonpareil’ genome sequence and whole-genome methylome data generated in this report represent a continued commitment of the *Prunus* community to improve the availability of genetic resources for these economically valuable species. Furthermore, this report presents the first whole-genome suite for ‘Nonpareil,’ the most widely grown almond cultivar in the United States and Australia. Access to these resources provides researchers in public and private institutions the ability to use genomics to improve and protect almond production, as well as address future challenges imposed by climate change, newly introduced pests or pathogens, and the volatility of consumer preference.

## Data availability

The raw sequencing data are available on NCBI Sequence Read Archive under the BioProject PRJNA769745. The genome assembly is available in the NCBI GenBank under the accession number JAJFAZ000000000. Supplementary files for the optical mapping are also available at NCBI under the SUPPF_0000004114. The transcriptome developed for this study was deposited in TSA with accession number: GJSC00000000, the version described in this paper is the first version, GJSC01000000.

Supplemental material is available at *G3* online.

## Supplementary Material

jkac065_Supplementary_File_1

## Notes

KMD-W coordinated the reception and shipment of samples, performed bioinformatics analyses, and wrote and revised the manuscript. TMG and GMS provided tissues samples and DNA/RNA samples and revised the manuscript. WZO and TM designed the bioinformatics pipeline for gene prediction and annotation and aided in the data analyses. JFR conceived, procured funding, designed, and coordinated the study as lead investigator, and participated in the data analyses, data deposit, and manuscript preparation, editing, and revision. The final manuscript has been read and approved by all authors.
