# Correction to: Whole-genome sequence and methylome profiling of the almond [*Prunus dulcis* (Mill.) D.A. Webb] cultivar ‘Nonpareil’

**DOI:** 10.1093/g3journal/jkac097

**Published:** 2022-05-09

This is a correction to: Katherine M. D’Amico-Willman, Wilberforce Z. Ouma, Tea Meulia, Gina M. Sideli, Thomas M. Gradziel, Jonathan Fresnedo-Ramírez, Whole-genome sequence and methylome profiling of the almond [*Prunus dulcis* (Mill.) D.A. Webb] cultivar ‘Nonpareil’, *G3 Genes|Genomes|Genetics*, 2022; jkac065, https://doi.org/10.1093/g3journal/jkac065

In the originally published version of this manuscript, the images for Figures 1 and 2 were swapped in error.

This error has been corrected.

