# Supplementary material for: Whole-genome sequence and methylome profiling of the almond [Prunus dulcis (Mill.) D.A. Webb] cultivar ‘Nonpareil’
Source: G3 (Bethesda). 2022 Mar 23;12(5):jkac065. doi: 10.1093/g3journal/jkac065 (PMC9073694; doi:10.1093/g3journal/jkac065)
Supplement: jkac065_Supplementary_File_1 [file jkac065_supplementary_file_1.docx]

**Pomological description of almond cultivars**

**Nonpareil** originated in Suisum, California in 1879 by nurseryman A.T. Hatch. It was selected from open-pollinated seed thought to be from the Languedoc region of France. The nut is considered to be of very high market quality, medium thick with a light color. Kernel/nut shelling percentage is typically 65% to 70% of nut having a poorly sealed paper shell. Kernel size averages 22-25 per ounce. The tree is upright-spreading, medium sized with consistently high yields. Nonpareil is very susceptible to Noninfectious Bud Failure.

**Texas**, also known as **Mission,** originated as a chance seedling in Texas at about 1891 and is also known by the synonyms Texas and Texas prolific. The nut is small, averaging about 25-28 kernels per ounce. The shell is medium hard and well-sealed with a shelling percentage of 55% to 65%. Mission harvest about 4 weeks after Nonpareil. The tree blooms late, typically about 1 week after Nonpareil and is productive with upright tree growth.

**Lauranne** originated in the INRA breeding program of Charles Grasselly in Avignon, France from a cross between Ferragnes and Tuono. It was introduced in 1989. The shell is very hard with a shelling percentage of about 30% to 40%. The kernels tend to be small and ovate and slightly wrinkled with a light color and a tendency to double ranging from 5% to 15%). Lauranne blooms late to very late, about 12 days after Nonpareil and harvests about 5 weeks after Nonpareil. The tree is spreading to drooping with medium vigor.

Adapted from: Brooks, R.M. and Olmo, H.P. (1997) Register of Fruit & Nut Varieties, 3rd edn. ASHS Press, Alexandria, Virginia.


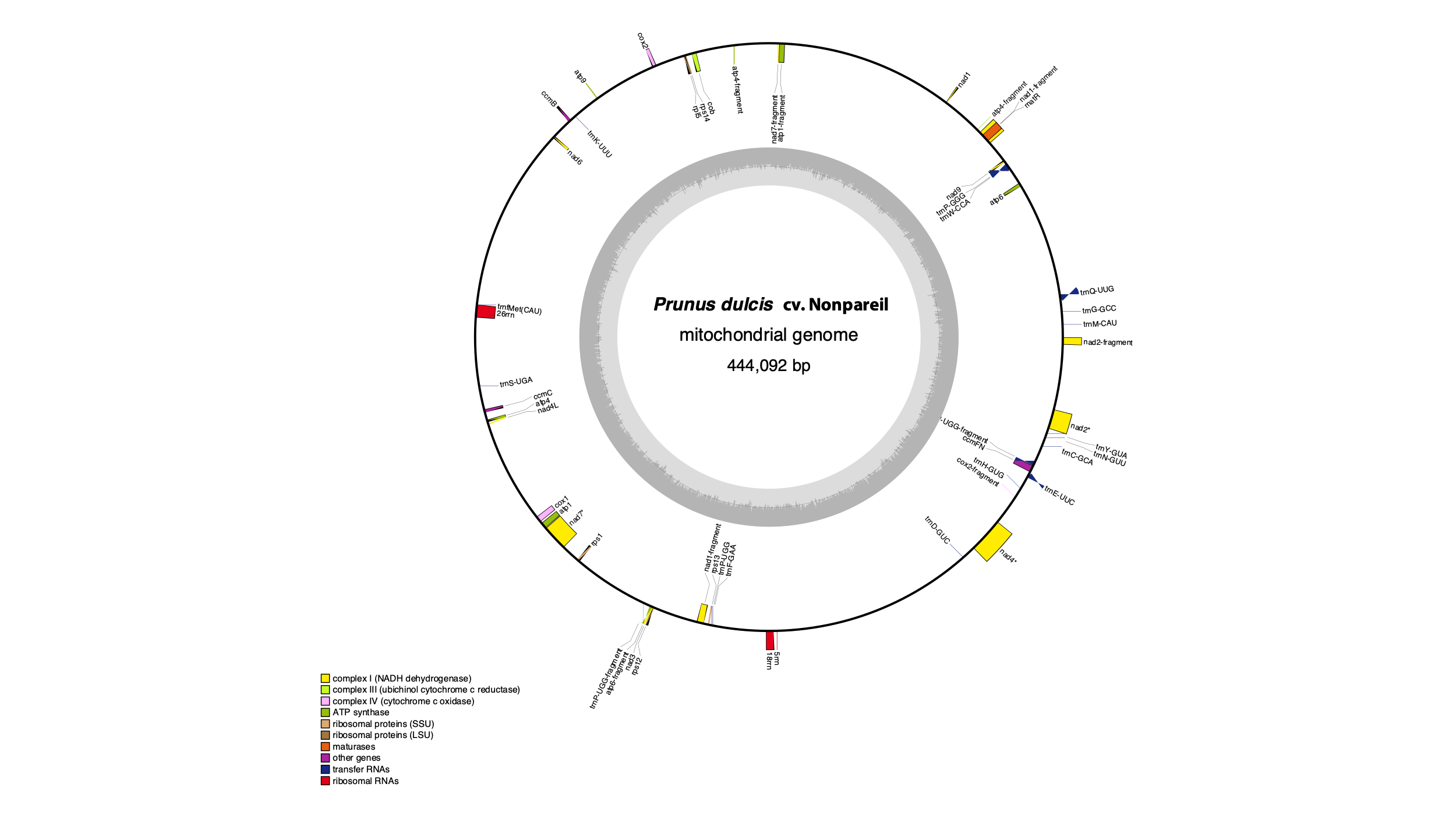


Fig. S1 Diagram of the plastidial genome of Nonpareil


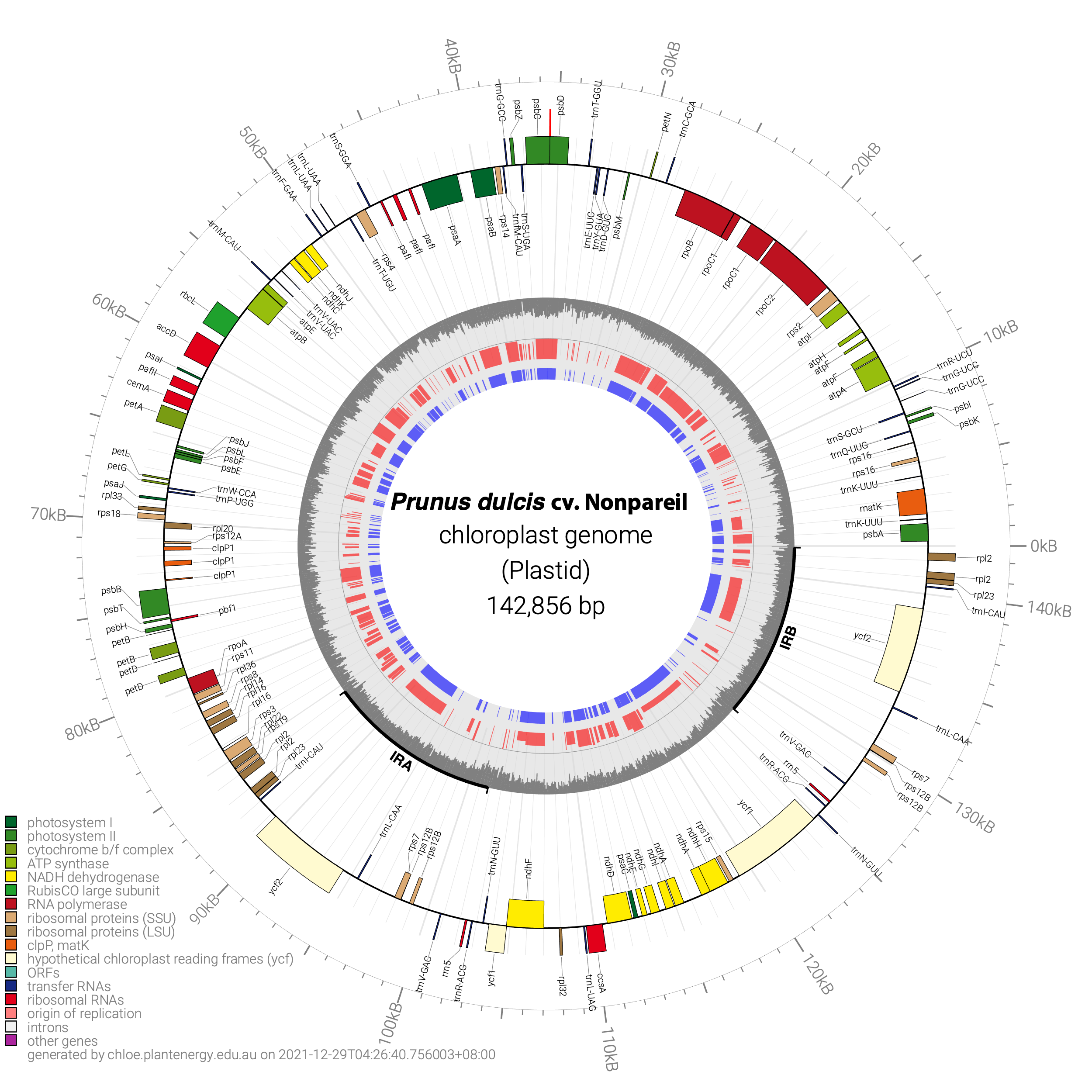


Fig. S2 Diagram of the mitochondrial genome of Nonpareil
